# Supplementary material for: Adherent cell remodeling on micropatterns is modulated by Piezo1 channels
Source: Sci Rep. 2021 Mar 3;11:5088. doi: 10.1038/s41598-021-84427-y (PMC7930019; doi:10.1038/s41598-021-84427-y)
Supplement: Supplementary file 6 — Supplementary Information. [file 41598_2021_84427_MOESM6_ESM.pdf]

Adherent cell remodeling on micropatterns is modulated by Piezo1 channels

Deekshitha Jetta[1,†], Mohammad Reza Bahrani Fard[1,†], Frederick Sachs[2], Katie Munechika[3], and Susan Z. Hua[1,2,*]

[1]Department of Mechanical and Aerospace Engineering, [2]Department of Physiology and Biophysics, [3]Department of Biomedical Engineering, University at Buffalo, Buffalo, New York 14260, USA

Supplemental Materials

Supplemental figures

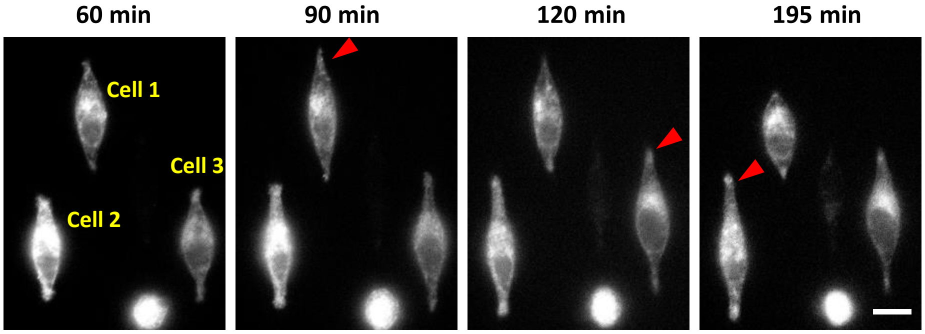

SM Figure 1. Time sequence of HEK-hP1 cell extending on the pattern. It shows that cells reached their maximum extension at different times. Red arrows indicate the cells in full elongation. Scale bar = 10 µm.

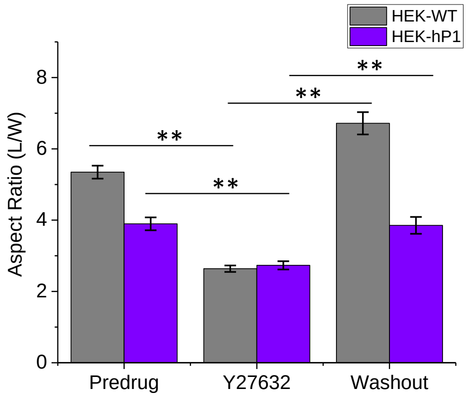

SM Figure 2. Effect of Rho-ROCK inhibitor on HEK-hP1 cells. Mean aspect ratio for pre-drug treatment, 20 min after treatment, and 60 min after washout for HEK-hP1 cells (n = 26, $**p < 0.001$) and control HEK cells (n = 60, $**p < 0.001$). It shows the effect of inhibitor was consistent in both HEK-hP1 and control cells.

Movies:

SM Movie 1. Expansion of control HEK cells on the stripes. Brightfield images of control HEK cells on the stripes showing cell expansion along pattern axis. The images were acquired at 3 min intervals from 60 min after seeding for a total recording time of 195 min.

SM Movie 2. Piezo1 knockout (P1KO) cells on the stripes. Brightfield images of P1KO cells on the stripes, showing these cells were not able to elongate along the pattern. The images were acquired at 3 min intervals from 60 min after seeding for a total recording time of 120 min.

SM Movie 3. Expansion of Piezo1 expressing (HEK-hP1) cells on the stripes. Brightfield images of HEK-hP1 cells on the stripes showing cell expansion along pattern axis. The images were acquired at 3 min intervals from 30 min after seeding for a total recording time of 150 min.

SM Movie 4. Translocation of Piezo1 plaques in hP1-HEK cells on the pattern. Fluorescence images (GFP) of hP1-HEK cells on the pattern, showing Piezo1 plaques translocate to expansion tips during cell elongation. The images were acquired at 3 min intervals from 60 min after seeding for a total recording time of 150 min.

SM Movie 5. Migration of a Piezo1 expressing cell on the pattern. Fluorescence images (GFP channel, in 16 color map) of a hP1-HEK cell on the pattern, showing Piezo1 plaques mainly located at the leading edge of the cell. The images were acquired at 3 min intervals from 120 min after seeding for a total recording time of 60 min.
